# Outbreak of acute hemorrhagic conjunctivitis in Yunnan, People's Republic of China, 2007

**DOI:** 10.1186/1743-422X-7-138

**Published:** 2010-06-25

**Authors:** Dongmei Yan, Shuangli Zhu, Yong Zhang, Jing Zhang, Yongming Zhou, Wenbo Xu

**Affiliations:** 1 WHO WPRO Regional Reference Poliomyelitis Laboratory and State Key Laboratory for Molecular Virology & Genetic Engineering, National Institute for Viral Disease Control and Prevention, Chinese Center for Disease Control and Prevention, 27 Nanwei Road, Beijing 100050, China; 2 Office for Disease Control and Emergency Response, Chinese Center for Disease Control and Prevention, 27 Nanwei Road, Beijing 100050, China; 3 Yunnan Center for Disease Control and Prevention, Dongshi Road, Kunming 650022, China

## Abstract

An outbreak of acute hemorrhagic conjunctivitis (AHC) occurred in Yunnan Province, China between August and September in 2007. A total of 3,597 cases were officially reported and the incidence rate reached 1390.94/100,000. Descriptive epidemiological analysis of the outbreak was conducted using the data from National Disease Supervision Information Management System (NDSIMS). To determine the causative agent for this outbreak and to analyze their genetic features, 30 conjunctival swabs and 19 paired serum specimens of acute and convalescent phase were collected from 30 patients with AHC, and viral isolation, molecular typing, antibody assay and phylogenetic analysis were performed.

11 virus strains were isolated from 30 conjunctival swabs. Amplification and sequencing of the VP4 region of these strains identified that coxsackievirus A24 variant (CA24v) could be the causative agent of the AHC outbreak and this was further confirmed by subsequent virus neutralizing antibody test on 19 paired serum specimens. Phylogenetic analysis based on the 3C regions showed that the Yunnan CA24v strains belonged to Group 3 and clustered with the strains isolated from worldwide AHC outbreaks after 2002. Phylogenetic analysis based on the partial VP1 revealed that the Yunnan strains differed from the strains isolated from AHC outbreak in Guangdong of China in 2007 with 2.8 - 3.0% nucleotide divergence, suggesting that two different lineages of CA24v caused the independent AHC outbreaks in Yunnan and Guangdong, respectively.

## Findings

Acute hemorrhagic conjunctivitis (AHC) is a highly contagious epidemic disease, commonly caused by Enterovirus 70 and Coxsackievirus A24 variant (CA24v). CA24v is an antigenic variant of the CA24 strain and was first isolated from an outbreak of AHC in Singapore in 1970 [1]. The AHC outbreaks caused by CA24v were restricted to Southeast Asia and India until 1985, and then spread rapidly to other regions of the world [2-4].

In China, the first outbreak of AHC was reported in 1971 [5]. Since then, outbreaks of AHC have been regularly reported [6,7]. Notifiable infectious diseases in China are classified into Class A, B, and C according to the degree of risk from the highest to the lowest. AHC belongs to Class C notifiable infectious disease. When a case is diagnosed as AHC, physicians are required to register the case of AHC to the National Disease Supervision Information Management System (NDSIMS), which is a direct reporting network system for notifiable infectious diseases in China. According to NDSIMS, a total of 74,263 AHC cases were reported in China in 2007, representing an increase of 540.58% compared with 11,593 cases in 2006. There were 134 reported outbreaks, spreading to 14 provinces and affecting 10,165 people. Other 64098 are sporadic cases. These data showed that a nationwide AHC epidemic occurred in China in 2007.

An outbreak of AHC occurred in Suijiang and its neighboring Shuifu Counties, Yunnan Province between August and September in 2007. In this study, descriptive epidemiological analysis was conducted using the data from NDSIMS and the causative agent was determined. 30 conjunctival swabs and 19 paired of acute and convalescent serum specimens were collected from 30 patients with AHC in Suijiang and Shuifu in September, 2007(Table 1). Two hundred microliter of the swab specimen was inoculated onto RD (rhabdomyosarcoma) and HEp-2 cells (human epidermoid carcinoma of the larynx). The cells were observed for 7 days for cytopathic effect (CPE). Two blind passages were performed when no CPE was observed.

**Table 1:** Epidemiological information and laboratory results of acute hemorrhagic conjunctivitis in Yunnan, China, 2007

					Date of acute and convalescent serum specimens collection		Virus isolation					
										
Case	Age (yr)/Sex	Occupation	Date of onset	Date of conjunctival swabs collection	Acute serum	Convalescent serum	RD	Hep2	Titre of acute serum against CA24v	Titre of convalescent serum against CA24v	4-fold or greater rise of antibodies titre	Amplification of the VP4 region
SJ1	7/F	Student	8 Sep. 2007	10 Sep. 2007	10 Sep. 2007	/*	Neg	Neg	/	/	/	Neg
SJ2	15/M	Student	9 Sep. 2007	10 Sep. 2007	10 Sep. 2007	30 Sep. 2007	Neg	Neg	<1:4	1:16	Yes	Neg
SJ3	38/F	Worker	10 Sep.2007	10 Sep. 2007	10 Sep. 2007	30 Sep. 2007	Neg	Pos	<1:4	1:64	Yes	Pos
SJ4	29/M	Worker	9 Sep. 2007	10 Sep. 2007	10 Sep. 2007	30 Sep. 2007	Neg	Pos	<1:4	1:16	Yes	Pos
SJ5	15/F	Student	10 Sep.2007	10 Sep. 2007	10 Sep. 2007	30 Sep. 2007	Neg	Pos	<1:4	1:64	Yes	Pos
SJ6	42/M	Worker	8 Sep.2007	10 Sep. 2007	10 Sep. 2007	/	Neg	Neg	/	/	/	Neg
SJ7	15/M	Student	9 Sep. 2007	10 Sep. 2007	10 Sep. 2007	30 Sep. 2007	Neg	Pos	<1:4	1:32	Yes	Pos
SJ8	14/F	Student	8 Sep. 2007	10 Sep. 2007	10 Sep. 2007	30 Sep. 2007	Neg	Neg	<1:4	1:32	Yes	Neg
SJ9	52/M	Worker	9 Sep. 2007	10 Sep. 2007	10 Sep. 2007	30 Sep. 2007	Neg	Pos	<1:4	1:64	Yes	Pos
SJ10	35/M	Worker	9 Sep. 2007	10 Sep. 2007	10 Sep. 2007	/	Neg	Neg	/	/	/	Neg
SF1	35/F	Farmer	9 Sep. 2007	9 Sep. 2007	9 Sep. 2007	29 Sep. 2007	Neg	Neg	<1:4	1:16	Yes	Neg
SF2	43/M	Farmer	7 Sep. 2007	9 Sep. 2007	9 Sep. 2007	29 Sep. 2007	Neg	Neg	1:4	1:16	Yes	Neg
SF3	46/F	Farmer	6 Sep. 2007	9 Sep. 2007	9 Sep. 2007	29 Sep. 2007	Neg	Neg	<1:4	1:32	Yes	Neg
SF4	45/M	Farmer	7 Sep. 2007	9 Sep. 2007	9 Sep. 2007	29 Sep. 2007	Neg	Neg	<1:4	1:128	Yes	Neg
SF5	33/F	Farmer	6 Sep.2007	9 Sep. 2007	9 Sep. 2007	29 Sep. 2007	Neg	Neg	<1:4	1:16	Yes	Neg
SF6	41/F	Farmer	6 Sep. 2007	9 Sep. 2007	9 Sep. 2007	29 Sep. 2007	Neg	Pos	<1:4	1:64	Yes	Pos
SF7	36/M	Farmer	5 Sep.2007	9 Sep. 2007	9 Sep. 2007	/	Neg	Neg	/	/	/	Neg
SF8	30/M	Farmer	6 Sep.2007	9 Sep. 2007	9 Sep. 2007	29 Sep. 2007	Neg	Neg	<1:4	1:16	Yes	Neg
SF9	35/M	Farmer	6 Sep. 2007	9 Sep.2007	9 Sep.2007	29 Sep. 2007	Neg	Pos	<1:4	<1:4	No	Pos
SF10	33/M	Farmer	9 Sep. 2007	9 Sep. 2007	9 Sep. 2007	/	Neg	Pos	/	/	/	Pos
SF11	14/M	Student	8 Sep. 2007	10 Sep.r 2007	10 Sep.r 2007	29 Sep. 2007	Neg	Neg	<1:4	<1:4	No	Neg
SF12	15/M	Student	9 Sep. 2007	10 Sep. 2007	10 Sep. 2007	29 Sep. 2007	Neg	Pos	<1:4	1:16	Yes	Pos
SF13	16/M	Student	9 Sep. 2007	10 Sep. 2007	10 Sep. 2007	29 Sep. 2007	Neg	Neg	<1:4	1:128	Yes	Neg
SF14	15/M	Student	8 Sep. 2007	10 Sep. 2007	10 Sep. 2007	29 Sep. 2007	Neg	Neg	<1:4	1:16	Yes	Neg
SF15	16/M	Student	8 Sep. 2007	10 Sep. 2007	10 Sep. 2007	/	Neg	Neg	/	/	/	Neg
SF16	18/M	Student	9 Sep. 2007	10 Sep. 2007	10 Sep. 2007	/	Neg	Neg	/	/	/	Neg
SF17	16/M	Student	9 Sep. 2007	10 Sep. 2007	10 Sep. 2007	/	Neg	Pos	/	/	/	Pos
SF18	18/M	Student	9 Sep. 2007	10 Sep. 2007	10 Sep. 2007	/	Neg	Pos	/	/	/	Pos
SF19	17/M	Student	8 Sep. 2007	10 Sep. 2007	10 Sep. 2007	/	Neg	Neg	/	/	/	Neg
SF20	17/M	Student	9 Sep. 2007	10 Sep. 2007	10 Sep. 2007	/	Neg	Neg	/	/	/	Neg

*the samples not available

Viral RNA was extracted from virus culture supernatant with a QIAamp Mini Viral RNA Extraction kit (Qiagen, Valencia, CA, USA). RT-PCR was performed using the primers OL68-1 and MD91 targeted for the VP4 region of the human enterovirus as described previously [8]. The obtained PCR products were purified with a QIAquick PCR Purification Kit (Qiagen, kk, Japan). The nucleotide sequence was determined by ABI PRISM 3100 Genetic Analyzer (Applied Biosystems, Hitachi, Japan) using BigDye Terminator Version 3.0 (Applied Biosystems, CA, USA). The serotype was determined by comparing the VP4 nucleotide sequence with those available at GenBank database using the BLAST program http://blast.ncbi.nlm.nih.gov/Blast.cgi from NCBI according to the criteria for molecular typing proposed previously [8].

The neutralizing antibody titers of the 19 paired serum specimens were determined by a virus neutralization assay. In brief, serial dilutions of the sera were mixed with 100 50% cell culture infective doses (CCID50) per 50 ul of the CA24v virus [Strain SF3, isolated from this outbreak, Genbank number EU596581 (VP1 region) and GQ229400(3C region)], incubated at 36°C for 2 hours and inoculated onto HEp-2 cells. A 4-fold or greater rise of CA24v neutralizing antibodies titre between acute and convalescent serum indicated an acute infection of CA24v.

For the molecular epidemiologic study, the partial VP1 and 3C region of Yunnan strains were amplified and sequenced with primers that flanked the VP1 region (forward, CVA24v-2407S: 5'-GTGAGTGCTTGCCCAGATTT-3'; reverse, CVA24v-3438A: 5'-ATACACCGCCATGTTCTGGT-3') designed in this study and primers 3C1/3C2 that flanked the entire 3C region [9]. The sequences of partial VP1 and 3C regions of the Yunnan isolates were aligned with those of the corresponding region of the different geographic and temporal CA24v strains using the CLUSTAL W method [10] in MEGA 4.0 [11]. Phylogenetic trees were constructed using the neighbor-joining method in MEGA 4.0 with bootstrap analysis performed on 1000 replicates. All sequences reported in this study were deposited into GenBank under accession number EU596577 to EU596589 and GQ229391 to GQ229410.

**Figure 1:**
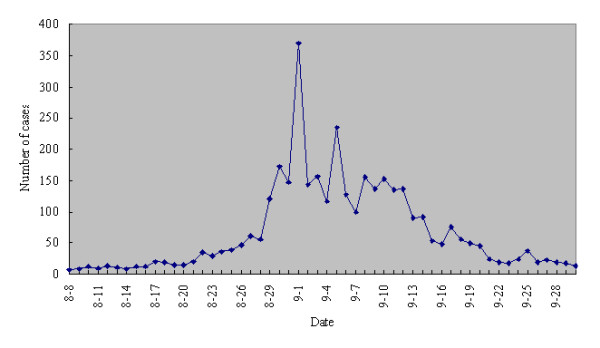
**Daily distribution of acute hemorrhagic conjunctivitis cases in Suijiang and Shuifu Counties, Yunnan in 2007**.

Shuifu and Suijiang Counties are adjacent counties located in the northeast of Yunnan Province and consist of 8 townships with a total population of 258,602 people. This AHC epidemic affected all 8 townships. By September 30, 2007, a total of 3,597 cases were officially reported by NDSIMS. The incidence rate reached 1390.94/100,000, no death and severe case occurred. Daily distribution of acute hemorrhagic conjunctivitis cases between August and September was shown in Figure 1.The first clinical diagnosed patient of the outbreak was identified on August 8, 2007 in Suijiang. Subsequently, the number of cases increased, reached a peak in the beginning of September and returned to baseline in late September (Figure 1). The age of the patients ranged from 3months to 84 years. The ratio between male and female was 1.71:1. Among all the patients, 46.4% were farmers, followed by students (31.9%) and the mean age was 37.7 years for farmers and 15.2 for students. The most common symptoms were conjunctival congestion (100%), followed by ocular stabbing pain (73.2%) and photophobia (64.6%). Some patients also appeared systemic symptoms such as fever (2.5%), fatigue (2.7%) and limbs pain (1.7%). 91.3% of all patients had a clear history of contact with people with similar symptoms.

As shown in table 1, HEp-2 cell was the only cell line capable of CA24v isolation in this study. Virus were isolated from 11(37%) of 30 swabs and VP4 regions were successfully amplified and sequenced for all 11 isolates (Table 1). All the sequences obtained have the highest identity (99.0-100.0%) with the CA24v strains (GenBank No. EU221286 to EU221294) isolated between July and October, 2007 in Guangdong Province of China [12], suggesting that CA24v could be the causative agent of the AHC outbreak. The virus neutralizing antibody analysis for 17 of 19 paired serum specimens showed a 4 fold or greater rise in neutralizing antibodies titer which provided further evidence that CA24v was the causative agent of the AHC outbreak in Yunnan (Table 1).

**Figure 2:**
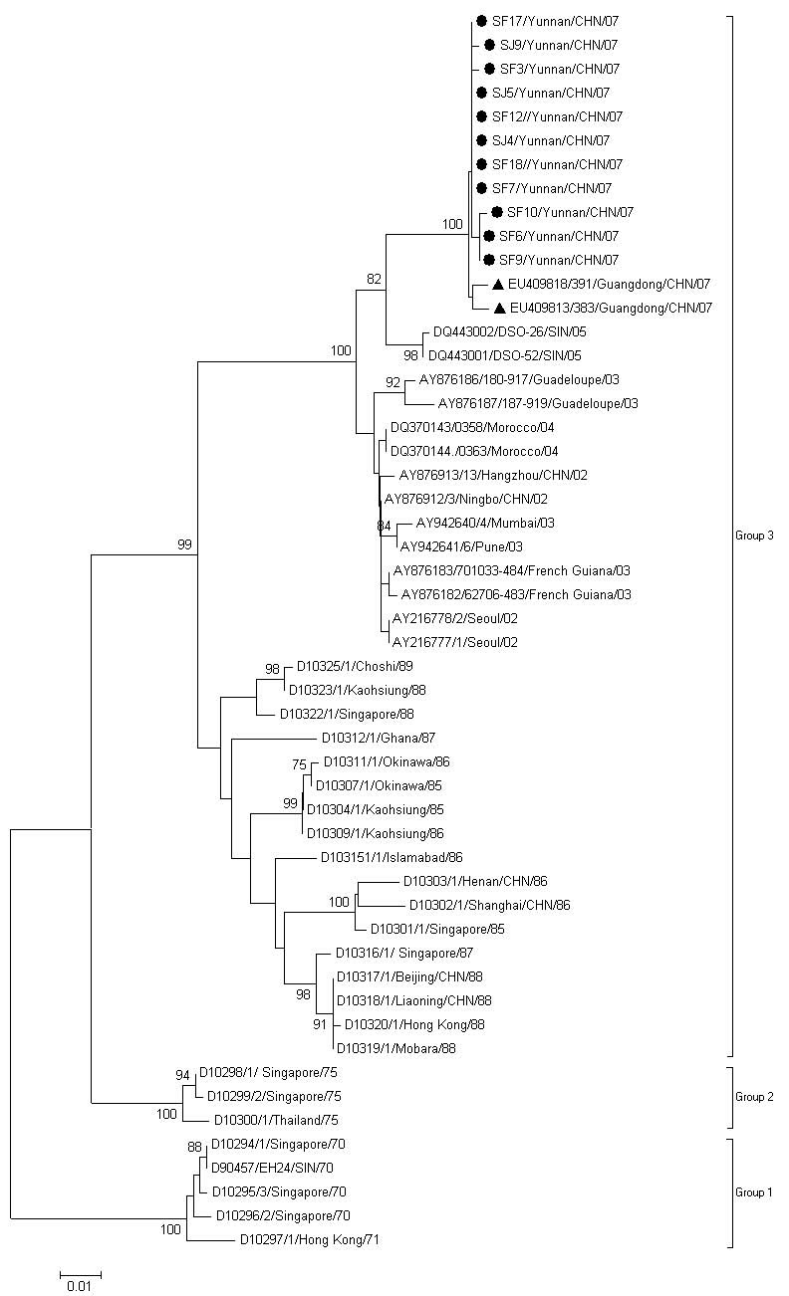
**Phylogenetic analysis based on the 3C region**. The nucleotide sequences of 3C region of Yunnan strains were compared with those of the CA24v strains from AHC outbreaks worldwide. In the unrooted tree obtained, Yunnan strains are indicated by black spots. Guangdong isolates were indicated by black triangle. The bootstrap values of at least 75% are shown at tree nodes. Every strain is indicated by GenBank accession number, strain code, country/region followed by year of isolation

**Figure 3:**
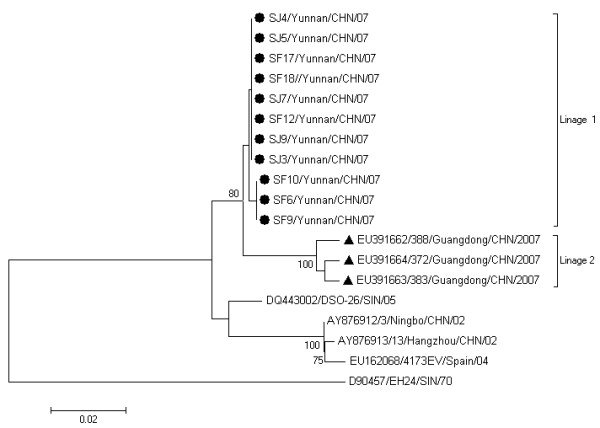
**Phylogenetic analysis based on the partial VP1 region**. The nucleotide sequences of partial VP1 region of Yunnan strains were compared with those of Guangdong strains and other CA24v strains with entire VP1 nucleotide sequence available in GenBank. The prototype CA24v strain EH24/70 was used as the root. Yunnan strains are indicated by black spots. Guangdong isolates were indicated by black triangle. The bootstrap values of at least 75% are shown at tree nodes. Every strain is indicated by GenBank accession number, strain code, country/region followed by year of isolation.

Similar to the previous studies [12,13], the phylogenetic analysis based on the 3C region revealed that all the CA24v strains were divided into three major groups. They are Group1 containing CA24v isolated from 1970 to 1971; Group 2, from 1975; Group 3, from 1985 to 2007. The Yunnan strains belonged to Group 3 and clustered with the strains isolated from worldwide AHC outbreaks after 2002 (Figure 2). The Yunnan strains were most closely related to those isolated in 2007 in Guangdong Province of China [12] with high nucleotide identities of 99.1 - 99.3% in 3C (Figure 2). In contrast to 3C region, the phylogenetic tree based on the partial VP1 region of CA24v strains showed more divergence between Yunnan and Guangdong strains, which clearly belonged to two different lineages (Lineage 1 and Lineage 2), respectively (Figure 3). The nucleotide identities within Lineages 1 and Lineages 2 were 99.7-100% and 98.6-99.2% respectively and A 2.8 -3.0% nucleotide divergence was found between these two lineages.

The outbreak of AHC described in this study occurred between August and September in Yunnan Province. The climate of this period in Yunnan is characterized by high temperatures and frequent rainfall, which may be two climatic factors involved in the rapid spread of CA24v strains. In this outbreak, the most patients were farmers (46.4%), while workers (37.56%) were the most predominant patient in the contemporaneous AHC outbreak in Guangdong [12]. That may be because farmers are the main population (accounting for 78% of the total number) in Shuifu and Shuijiang Counties of Yunnan. Moreover, August and September are in the rice harvest season and local farmers have the habit of laboring together, resulting in exposure time and frequency increased significantly. In addition, in rural areas such as these two counties, the phenomenon of sharing the basins and towels was very common which played an important pole in causing the outbreak.

Our study demonstrated that CA24v was the causative agent of this AHC outbreak in Yunnan, southern China. Moreover, AHC outbreaks reported in Guangdong (southern China) [12] and Beijing (northern China) [14] in 2007, were also caused by CA24v. Although the information on the causative agent of AHC outbreaks in other provinces was not obtained, it is possible that CA24v was major causative agent during the national AHC outbreak considering the close temporal and geographical relation.

Although 3C region was commonly used for molecular epidemiological analysis of CVA24v strains, recent studies have demonstrated that capsid encoding region may be more informative than the 3C region because of the high recombination rate in nonstructural region between enteroviruses and serological pressure on the capsid encoding region which therefore may evolve faster than the region encoding for the nonstructural proteins. In this study, compared with 3C region, phylogenetic tree based on the partial VP1 region of CA24v strains showed more divergence between Yunnan and Guangdong strains and clearly differentiated Yunnan lineage and Guangdong linage, indicating that two independent outbreaks occurred in Yuannan and Guangdong although the AHC outbreaks in these two provinces was temporally and geographically related.

## Abbreviations

AHC: acute hemorrhagic conjunctivitis; CA24v: coxsackievirus A24 variant; RT-PCR: Reverse transcription-polymerase chain reaction.

## Competing interests

The authors declare that they have no competing interests.

## Authors' contributions

Dongmei Yan and Shuangli Zhu performed the experiment. Jing Zhang and Yong-ming Zhou had made substantial contributions to acquisition of epidemiological information. Dongmei Yan drafted the manuscript. Yong Zhang and Wenbo Xu revised the manuscript. All authors read and approved the final manuscript.
